# First in-Lab Testing of a Cost-Effective Prototype for PM_2.5_ Monitoring: The P.ALP Assessment

**DOI:** 10.3390/s24185915

**Published:** 2024-09-12

**Authors:** Giacomo Fanti, Francesca Borghi, Cody Wolfe, Davide Campagnolo, Justin Patts, Andrea Cattaneo, Andrea Spinazzè, Emanuele Cauda, Domenico Maria Cavallo

**Affiliations:** 1 Department of Science and High Technology, University of Insubria, Via Valleggio 11, 22100 Como, Italy; davide.campagnolo@uninsubria.it (D.C.); andrea.cattaneo@uninsubria.it (A.C.); andrea.spinazze@uninsubria.it (A.S.); domenico.cavallo@uninsubria.it (D.M.C.); 2 Department of Medical and Surgical Sciences, University of Bologna, Via Palagi 9, 40138 Bologna, Italy; francesca.borghi12@unibo.it; 3 Center for Direct Reading and Sensor Technologies, National Institute for Occupational Safety and Health, Centers for Disease Control and Prevention, Pittsburgh, PA 15236, USA; yvv5@cdc.gov (C.W.); jdq7@cdc.gov (J.P.); cuu5@cdc.gov (E.C.)

**Keywords:** miniaturized monitors, aerosol chamber, air quality, air pollution, exposure assessment, low-cost monitor

## Abstract

The goal of the present research was to assess, under controlled laboratory conditions, the accuracy and precision of a prototype device (named ‘P.ALP’: Ph.D. Air-quality Low-cost Project) developed for PM_2.5_ concentration level monitoring. Indeed, this study follows a complementary manuscript (previously published) focusing on the in-field evaluation of the device’s performance. Four P.ALP prototypes were co-located with the reference instrument in a calm-air aerosol chamber at the NIOSH laboratories in Pittsburgh, PA (USA), used by the Center for Direct Reading and Sensor Technologies. The devices were tested for 10 monitoring days under several exposure conditions. To evaluate the performance of the prototypes, different approaches were employed. After the data from the devices were stored and prepared for analysis, to assess the accuracy (comparing the reference instrument with the prototypes) and the precision (comparing all the possible pairs of devices) of the P.ALPs, linear regression analysis was performed. Moreover, to find out the applicability field of this device, the US EPA’s suggested criteria were adopted, and to assess error trends of the prototype in the process of data acquisition, Bland–Altman plots were built. The findings show that, by introducing ad hoc calibration factors, the P.ALP’s performance needs to be further implemented, but the device can monitor the concentration trend variations with satisfying accuracy. Overall, the P.ALP can be involved in and adapted to a wide range of applications because of the inexpensive nature of the components, the small dimensions, and the high data storage capacity.

## 1. Introduction

### 1.1. Background

Air pollution is a major environmental risk factor for global health and the fourth risk factor for global mortality [1] and remains one of the main concerns in most European territories, especially in urban areas [2]. Potential health effects associated with air pollution exposure include cardio-cerebrovascular outcomes, decreased lung function, aggravation of respiratory diseases, and increased asthma incidence and severity, among a variety of other effects [3,4]. In early 2020, analyses conducted by scientists across Europe due to the COVID-19 pandemic showed that exposure to air pollution can influence humans’ vulnerability and susceptibility to the disease [2,5,6]. For implementing air policies, air-quality monitoring data with a high spatial and temporal resolution are needed [7]. In recent years, the needs of the scientific, industrial, and civilian communities have changed and are evolving under the scientific research point of view introducing new particulate matter (PM) monitoring methods and techniques like spectroscopy detection [8] and polarization lidar [9]. It should be necessary that the reference instrument combines side by side monitoring with the abovementioned needs, but it is still lacking regarding several aspects (e.g., costs, spatiotemporal resolution, portability, and power consumption) and, nowadays, it cannot fill these gaps by itself. This situation has highlighted an increasing need for new instrumentation, which could accelerate the improvement of air-quality monitoring science and project the results for academics and professionals. Pairing the reference techniques with recent technologies and devices could allow an improvement of data quality and/or quantity [7,8,9,10,11,12,13]. These instruments, when compared to the traditional ones, are generally light and small, are characterized by low power consumption, and require less maintenance and handling compared to the reference devices [14].

### 1.2. Problem Statement

The recent emergence of low-cost sensor technologies has opened new possibilities in air-quality monitoring activities [15,16] while introducing new challenges regarding their usage, performance, and applicability. Several manufacturers are producing and commercializing a wide range of devices and instruments, which have been classified and cataloged in a recent publication [17] as Next-Generation Monitors and Sensors (NGMSs). Furthermore, Plantower (PMS3003; PMS5003) and Sharp Electronics (GP2Y1010AU0F) are among the most-used sensors for the measurement of size-fractioned PM concentrations. These light-scattering sensors are known to be subjected to several factors that could affect their performance (i.e., temperature, relative humidity, and type of dust) [17]. All these commercially available devices still have different costs, usability, and applicability in the field of air-quality monitoring and investigation. The results of several studies, both in laboratories and in the field, on the evaluation of the performance of NGMSs have been published in recent years [18,19,20,21,22,23]. These publications mainly focused on assessing the performances of these instruments (mainly in terms of precision and accuracy) but, even if the latter must be the first step approaching these innovative technologies, a concrete discussion on their possible contribution in exposure science topics is lacking. Furthermore, there is a constant need for improved technologies and the possibility of having a device that can be personalized following the specific needs of end-users.

### 1.3. Aim of the Study

To fill the lack of available devices able to be paired to reference instruments, a homemade, low-cost (LC), and customizable prototype was designed and assembled at Insubria University (based in Como, Italy) [24]. The abovementioned device has been named P.ALP (Ph.D. Air-quality Low-cost Project) and it was previously tested under non-controlled conditions in four different plausible environments (i.e., office, home, outdoor, and occupational) [25]. In previous tests [25], it has been found that, in real working conditions, the P.ALP prototype’s applicability field should be at medium–low PM_2.5_ concentrations (<30.87 µg/m^3^) in both indoor and outdoor environments. In this complementary manuscript, the P.ALP assessment was carried out under controlled conditions, and it was exposed to varying types of dust, dust concentrations, temperatures, and relative humidity using a Marple Dust Chamber [26]. This study aims to evaluate the performances, specifically in terms of precision and accuracy [27,28], of the device compared to the reference instrument. This study also focuses on observing the P.ALP in its best application field following the guidelines for the evaluation of low-cost PM sensors [23]. Furthermore, this study aims to, once the performances of the device are assessed, open a discussion on the possibilities raised by this prototype and similar ones, regardless of their agreement with the quality standards and performances guaranteed by the reference instrument.

## 2. Materials and Methods

### 2.1. Instruments and Setup

All the technical features regarding the device assessed in this manuscript are reported in a previous publication [24]; hereafter, only its main characteristics strictly needed for the scope of the present manuscript are reported. The P.ALP, in its to date (November 2023) setup, can be used to collect information on (i) PM concentrations (PM_1_, PM_2.5_, and PM_10_), (ii) relative humidity (RH), and (iii) temperature (T). The Plantower PMS5003 sensor, which provides the PM data, consisted of a particle concentration sensor whose working principle is based on light-scattering technology, and it operates at 0.1 L/min. The latter can provide the number of airborne particles suspended in the air and it is able generate an output via a digital interface, collecting concentration data over time [24]. The T and RH data were acquired by the sensor DHT22 (also called AM2302), which provided, through digital signals, data on the ambient temperature and relative humidity using a thermistor and a capacitive humidity sensor to collect information about the air that surrounded it [24]. The P.ALP can be power-supplied by any commercial power bank (e.g., a 10,000 mAh battery can satisfy the power demand of the device for at least 48 h), and the data collected were stored on a MicroSD card. Four devices (i.e., P.ALP_0; P.ALP_1; P.ALP_2; and P.ALP_3) were built with the same characteristics and configurations to assess the precision of the P.ALP’s design. The reference data were acquired through a reference instrument (i.e., TEOM 1400a—Thermo Fisher Scientific, Waltham, MA, USA), equipped with a Dorr-Oliver cyclone operated at 1.7 L/min, which provided an acceptable approximation of PM_2.5_ 15 min-averaged concentrations. A laboratory-grade aerosol particle sizer (APS, TSI, model APS3321), with an acquisition rate of 1 datapoint per minute, was co-located inside the chamber and connected to a computer outside the chamber. Briefly, the intercomparison tests provided a ratio between the results of PM_2.5_ concentrations through TEOM and APS (6.32 ± 1.13). These correction factors (mean ± SD) were then provided, to be applied a posteriori to the PM_2.5_ data obtained through APS for both granite mine dust (5.70 ± 1.35) and gold mine dust (6.69 ± 0.65). Experimentation was conducted using an aerosol calm-air chamber at the laboratory of the National Institute for Occupational Safety and Health (NIOSH) in Pittsburgh (PA), USA. The chamber (dimensions: (i) hexagonal cross-section was approximately 2.4 m high with (ii) an inside effective diameter of 1.2 m and (iii) an internal volume of 1.8 m^3^) can be used to simultaneously test several devices side by side, and it provides an internal spatial variability less than 5%. The chamber was fed with filtered air by a mass flow, humidity, and temperature controller (Miller Nelson; model HCS-501; Livermore, CA, USA). Separately, a fluidizer bed aerosolizer (TSI 3400A) mixed additional air with the pre-loaded dust, and this mixture entered a radioactive neutralizer (TSI 3012A NCR). The mixture of air and dust entered the chamber from the top, passing through a honeycomb flow straightener. Lastly, the air was drawn down to the bottom of the chamber by a vacuum system, which can be controlled using a damper. Two different dusts, collected in active workplace operations and sieved in preparation for use in the calm-air chamber, were used for five days each: (i) Dust 1—granite mine dust (GrMD; approximate composition: plagioclase 34%, K-feldspar 36%, and quartz 30%) and (ii) Dust 2—gold mine dust (GoMD; approximate composition: plagioclase 20%, chlorite 29%, muscovite 25%, dolomite 4%, and quartz 22%).

### 2.2. Data Collection

The data were acquired through 10 data-collecting days performing a total of 20 different tests as summarized in Table 1, which reports the planned schedule of the entire testing process.

Each test was conducted to challenge the sensors under a wide range of conditions and dust concentrations. Due to the characteristics of the fluidizer bed aerosol generator (TSI 3400A) used for varying the dust concentrations inside the chamber environment, it was not possible to preselect the exact level of PM_2.5_ desired, but it was only possible to plan the range of concentrations at which conduct the tests as shown in Table 1. The sessions were different from each other to reproduce, inside the chamber, realistic (i) extremely variable dust conditions (spikes), (ii) steady-state dust concentrations, (iii) RH variations, (iv) constantly increasing dust concentrations, and (v) a sum of all the previous situations reproduced randomly. The tests’ duration was determined using a filter-based technique, co-located inside the chamber, whose results were not included in this manuscript due to its specific aims (P.ALP performance assessment) already declared. During the experiments, it was not possible to directly control the temperature, but it was constantly monitored. To increase the variability in these experiments, two different types of dusts were used. The first five days of testing were conducted using Dust 1-GrMD, and from day 6 to day 10 we used Dust 2-GoMD. Between each test, the chamber was cleaned, flushing only fresh air to lower the dust concentrations until the reference instrument (inside the chamber) detected levels comparable to the lab’s ambient environment. Furthermore, by cleaning the chamber, we created the conditions to be able to conduct independent tests.

### 2.3. LOD and LOQ

This study was the very first to test the P.ALP prototype under controlled conditions, so calculations of the limit of quantification (LOQ) and limit of detection (LOD) were performed. The standard deviation of the blank method [29] was used to estimate the LOD of the P.ALP:

$$LOD=\frac{3\sigma_{blk}}{k}$$

where σ_blk_ is the standard deviation of the sensor output in blank conditions and k is the slope of the linear relationship of the P.ALPs’ measurement values versus the reference (APS) during the same conditions, considering the entire dataset. In this manuscript, the authors adopted the same approach reported by Sayahi et al., 2019 [22], that is, to consider as blank measurements the PM_2.5_ data acquired by the P.ALPs when the reference instrument, the APS, acquired PM_2.5_ concentrations lower than 1 µg/m^3^. The LOQ of the P.ALP was calculated as indicated by the Reg. (EU) 333/2007 [Eq. D] [30]:

LOQ=3.3 × LOD

Because this was the very first in-lab assessment, under controlled conditions, of the prototype’s performance, the authors decided to keep the data lower than the P.ALP’s LOD values for the whole data assessment and statistical analysis process to preserve the information regarding very-low concentrations and to be able to evaluate it. Moreover, because the LODs and the LOQs of the APS are expected to be lower than the P.ALP’s, the values below the latter do not necessarily characterize a condition below the lowest LOD and the LOQ (APS) visible in the chamber environment.

### 2.4. Data Treatment and Statistical Analysis

At the end of each test, raw data acquired by the prototypes were reorganized by R script (R Statistical Software (v4.1.2; R Core Team 2021)), freely available in [24]. Data clearly affected by hardware issues were identified and removed from the database (DB) (i.e., P.ALP_3 day 1 and day 2). The percentiles (i.e., 33rd; 66th) were calculated on the entire dataset to be able to categorize the DB into three different concentration ranges (low concentrations, mean concentrations, and high concentrations) to assess the prototypes’ performance in every single working condition investigated. The data acquired by TEOM 1400a and by the APS were averaged day by day to calculate specific correction factors. The latter was adopted to correct the APS values and thus justify the consideration, as a reference value for PM_2.5_ concentrations, those data, characterized by a 1 datapoint-a-minute time resolution. Moreover, concerning the APS, data up to the aerodynamic diameter of 2.458 µm were used to estimate the PM_2.5_ concentrations. This was later adopted as high-time-resolution reference data to conduct prototype evaluations. A *p*-value lower than 0.05 was assumed, for all tests, as statistically significant. Regarding (i) the reference instrument (APS), (ii) the four P.ALPs, (iii) RH, and (iv) T acquired data, a descriptive statistics analysis was performed concerning the PM_2.5_ concentrations. A Kolmogorov–Smirnov test was conducted on the entire dataset aiming to explore the data distribution, and the result was a non-normal distribution. Moreover, after a log transformation, the data were non-normally distributed. Consequently, when evaluating all the possible pairings of P.ALPs, the non-parametric Mann–Whitney test was conducted for assessing the differences between the data obtained from paired P.ALPs. The performance of the prototypes was evaluated by carrying out several tests: (i) a linear regression analysis was adopted to assess the precision of the devices following the criteria published by Watson et al. (1998) [27] by conducting comparability evaluations between each pair of tested prototypes and the predictability of one device compared to that of another. (ii) Considering the reference measurement (APS—i.e., the independent variable) versus the investigated devices (the four prototypes—i.e., the dependent variable), using the abovementioned method, we evaluated the accuracy of the prototypes. Adopting this approach, two measurement techniques can be assumed comparable, if R > 0.9 (where R is the correlation coefficient). Furthermore, if R > 0.9, the intercept is equal to 0 (±3 × standard error) and the slope is equal to 1 (±3 × standard error). The two measurement techniques can also be considered reciprocally predictive [27]. Precision (confrontation between the P.ALPs) and accuracy (confrontation between the APS and the prototypes) assessments were conducted on the whole dataset. Thereupon, in accordance with the US EPA air-sensor guidebook [3,20,23], the mean normalized bias (MNB) and coefficient of variation (CV) of the data collected by the devices were assessed to find a proper application field for PM_2.5_ monitoring for each tested prototype. We adopted the theoretical bases suggested by Williams et al. (2014) [23]: (i) Tier I: (−0.5 < MNB < 0.5 and CV < 0.5) ‘Education and Information’; (ii) Tier II: (−0.3 < MNB < 0.3 and CV < 0.3) ‘Hotspot Identification and Characterization’; (iii) Tier III: (−0.2 < MNB < 0.2 and CV < 0.2) ‘Supplemental Monitoring’; (iv) Tier IV: (−0.3 < MNB < 0.3 and CV < 0.3) ‘Personal Exposure’; and (v) Tier V: (−0.1 < MNB < 0.1 and CV < 0.1) ‘Regulatory Monitoring’. Using Bland–Altman plots [31], the assessment of the possible error trends of the prototypes was performed. The latter graphs report the absolute deviation between the instrument (P.ALP) and the results of the compared reference instrument (APS) for each pair of measurements, which were obtained using session average data. An evaluation of the average errors and the related lower and upper 95% confidence intervals (95% CI) was also conducted. Furthermore, the data acquired by the P.ALPs were time-weighted average (TWA)-corrected by the TEOM to show the influence of post-calibrations on our prototype. The data collected were statistically analyzed using the software SPSS Statistics 20.0 (IBM, Armonk, NY, USA).

## 3. Results

An total of 10 sessions of monitoring (a total of circa 50 h of testing) was conducted between September and October 2022. In Table 2, an overview of the dust-chamber environment values that characterized the testing sessions is reported. All the PM_2.5_ data presented in Table 2 were acquired by the APS and were corrected based on the TEOM data. Reported data concerning RH% and T were acquired by the four P.ALPs; instead of reporting the mean RH% values, we provided the RH range in which the Plantower PMS5003 sensor was evaluated, aiming to make sure it fell within its nominal functioning RH% range (0~99%). RH data, together with the average T data (the temperature inside the chamber cannot be set and controlled), are used in this study only as contextual data. The effects of temperature and relative humidity on the performance of the P.ALP will be explored in future studies.

The following paragraphs present the outcomes of the experiments obtained by the abovementioned data analysis process.

### 3.1. Descriptive Statistics

The LOD and LOQ values were calculated following the approaches described in Section 2.3, and their values were 2.12 µg/m^3^ and 6.99 µg/m^3^, respectively. Because this was the very first assessment of the P.ALP prototype, the authors decided to include all the <LOD data into the following statistical analysis process to include any possible information regarding the performances of the devices also at very low concentrations. Furthermore, the decision was made to even include the data acquired during the so called ‘sensitivity tests’ conducted on day 1 and day 6. Table 3 presents the PM_2.5_ concentrations’ summary statistics, expressed in µg/m^3^, collected by the four prototypes and the APS (reference instrument).

### 3.2. Precision

To assess the precision of the P.ALPs, as already declared in Section 2.3, each pair of co-located devices was investigated through the linear regression analysis approach adopting Watson et al.’s criteria [27] to assess if they are comparable, mutually predictable, or neither (Table 4). Hereafter are presented the results from the analysis of the whole dataset, but the analyses were also performed by dividing the dataset by (i) concentration range (Table S3) and (ii) type of dust (Table S4).

As shown in Table 4, when performing this analysis including the entire dataset, the R coefficient outcomes are always higher than 0.9, which, as stated before, is the minimum criterion for the comparability of two devices following Watson et al., 1998 [27]. From this, the four prototypes can be considered as always comparable to each other. In contrast, the devices cannot be considered reciprocally predictive, except for the paired devices P.ALP_1 and P.ALP_3.

### 3.3. Accuracy

The assessment of the accuracy of the prototypes was conducted in the same way as that regarding the precision evaluation, by applying the Watson et al.’s (1998) guidelines [27]. The co-located reference instrument (APS) was compared against the data collected from P.ALPs. Table 5 presents the outcomes obtained from the accuracy analysis.

### 3.4. Application Field following US EPA’s Guidelines

Aiming to place the P.ALPs in their most suitable applicability field, the US Environmental Protection Agency’s guidelines [23] were adopted. Following this, Table 6 presents the results of this analysis.

### 3.5. Error Trends

**Figure 1:**
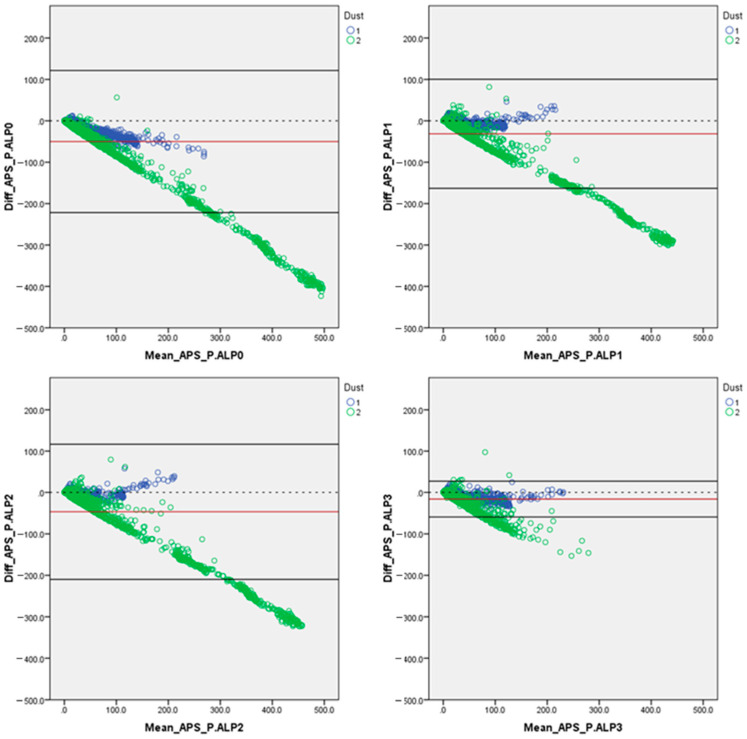
Bland–Altman plots for the data collected by the four prototypes plotted against the reference instrument (APS); both X and Y axes are reported in [µg/m^3^]. In blue are highlighted the data referring to the GrMD and in green are highlighted the data referring to GoMD. The dotted black line represents the theoretically perfect agreement between the two compared devices (APS and P.ALP). The solid red line indicates the mean error between the two techniques compared, and the two solid black lines highlight the upper and the lower 95% confidence intervals.

The Bland–Altman plot method [31] was performed to deeply investigate any possible error trends of the P.ALPs under evaluation. In this section, the statistics of the error trends are presented (Table 7), as well as the main outcomes of the evaluations performed considering the entire dataset (Figure 1). However, like we performed for the previously presented evaluations, also in this case, the data were investigated by splitting the dataset into dust (graphically) and concentration range. The latter analyses are presented in the Supplementary Materials in this manuscript (Figures S1–S3).

#### Error Trends of the P.ALP’s Post-Corrected Data

To further compare the data acquired form the two different instruments’ typologies (APS and P.ALP), at this point, all the data were TWA-corrected by the TEOM’s data to handle them following the same approach and evaluate the influence of the application of a post-correction factor on the P.ALP. The statistics of the error trends of this latter analysis are presented in the following Table 8.

**Table 8:** Statistics of the average error trends used for building the Bland–Altman plots, presented in Figure 2. Mean: mean of the whole dataset used in this assessment; SD: standard deviation value.

Devices Compared	PM_2.5_ Average Error [µg/m^3^]		PM_2.5_ Confidence Interval [µg/m^3^]	
	Mean	SD	Upper 95%	Lower 95%
APS vs. P.ALP_0	1.02	5.61	12.01	−9.98
APS vs. P.ALP_1	−2.21	9.43	16.40	−20.81
APS vs. P.ALP_2	3.68	16.77	36.54	−29.18
APS vs. P.ALP_3	1.91	8.44	18.46	−14.63

## 4. Discussion

### 4.1. Descriptive Statistics

Concerning the results for the analysis of the entire dataset (Table 3 and Figure 3), the mean values regarding the PM_2.5_ concentrations (mean ± S.D.) of the prototypes differ from APS’s. P.ALP_3 is the closest prototype, on average, to the reference instrument (APS), but this is because, during the second day of testing, it was faulty when the ‘high-concentrations’ test was conducted. Furthermore, on average, overestimated PM_2.5_ concentrations were obtained with the P.ALPs.

In Tables S1 and S2, the results of the descriptive statistics of the dataset split by concentration range and dust are presented. The results from the statistical analysis conducted on the dataset split into different dusts (Figure 4 and Table S2) highlight a better performance, on average (mean ± S.D.), of the prototypes versus the reference instrument with the first dust (GrMD) instead of the second (GoMD).

Furthermore, when evaluating the outcomes form the statistical analysis of the dataset split by concentration range presented in Figure 5 and Table S1, it is possible to assert that, on average (mean ± S.D.), the P.ALPs compared to the APS performed better at low (<9.39 µg/m^3^) and mean (9.39 < x < 45.97 µg/m^3^) PM_2.5_ concentrations than at higher ones (>45.97 µg/m^3^).

### 4.2. Precision

By adopting Watson et al.’s criteria [27], performing the evaluations on the whole dataset, it was possible to conclude that all the prototypes were comparable, but only P.ALP_1 and P.ALP_3 resulted in being mutually predictable (Table 4). By splitting the dataset by concentration range, the outcome of the evaluations was almost the same because all the prototypes showed comparability, and only in a few cases was mutual predictability highlighted, as reported in Table S3. Moreover, by splitting the dataset by dust, all the prototypes were compared to each other, and only in three cases were they also mutually predictable, as show in Table S4.

### 4.3. Accuracy

Focusing on the results highlighted in Table 5, evaluating the entire dataset, it is possible to declare a comparable situation between the reference instrument (APS) and all four prototypes. As expected, due to the gap in terms of the performance level between a prototype and a reference instrument, a situation of non-mutual predictivity was highlighted considering the P.ALPs and the APS. Further evaluations were conducted to deeply investigate the accuracy of the P.ALPs in several conditions and to show that the regression analysis was handled by splitting the dataset by concentration range and by dust. The results of the abovementioned analysis are presented in Tables S5 and S6.

### 4.4. US EPA’s Guidelines

From the US EPA’s guidelines on Air Sensors [3,23], it was possible to define the applicability domain of the P.ALP prototype based on the environment in which it is used. Firstly, the evaluation was performed on the whole dataset and, as shown in Table 5, only P.ALP_3 was suitable to be considered useful in one of the suggested tiers. To more thoroughly investigate the outputs of the prototypes, an identical approach was used, splitting the database by concentration range; the results are shown in Table S7 (Supplementary Materials). The investigation was also performed by splitting the dataset by dust, and what emerged was that the performances of the prototypes were better with the first dust (GrMD) than with the second one (GoMD); the results are reported in Table S8 (Supplementary Materials). The dataset was further investigated with the same approach, by splitting it by concentration range and dust simultaneously, and it was confirmed that the prototypes performed better with the GrMD than with the GoMD, as reported in Table S9 (Supplementary Materials).

### 4.5. Error Trends

The Bland–Altman plots presented in Figure 1 show, taking into account the whole dataset, the same error trends regarding all prototypes. In fact, the P.ALPs showed a good agreement between the two techniques we compared at lower concentrations, but they revealed an increasing overestimation trend with an increasing concentration. The accordance between the two techniques compared is stronger at low and medium PM_2.5_ concentrations because the error trend of overestimation increased with the increase in the concentration of the dust inside the chamber. Anyway, it is clearly shown that, on average, all four tested prototypes are affected by an overestimation of the considered PM_2.5_ concentrations (acquired by the reference instrument). Overall, after the data analysis, it can be stated that the prototypes showed better performances with the GrMD instead of the GoMD (Figures S1–S3—Supplementary Materials). This could be because different dusts might have different light-scattering properties related to specific dusts’ characteristics (e.g., size distribution, hygroscopicity, and density) [32], and because the working principle of the PM sensor (PMS5003) is based on light scattering [22,33], it can affect the performances of the investigated prototype.

### 4.6. Error Trends—P.ALPs’ Post-Corrected Data

**Figure 2:**
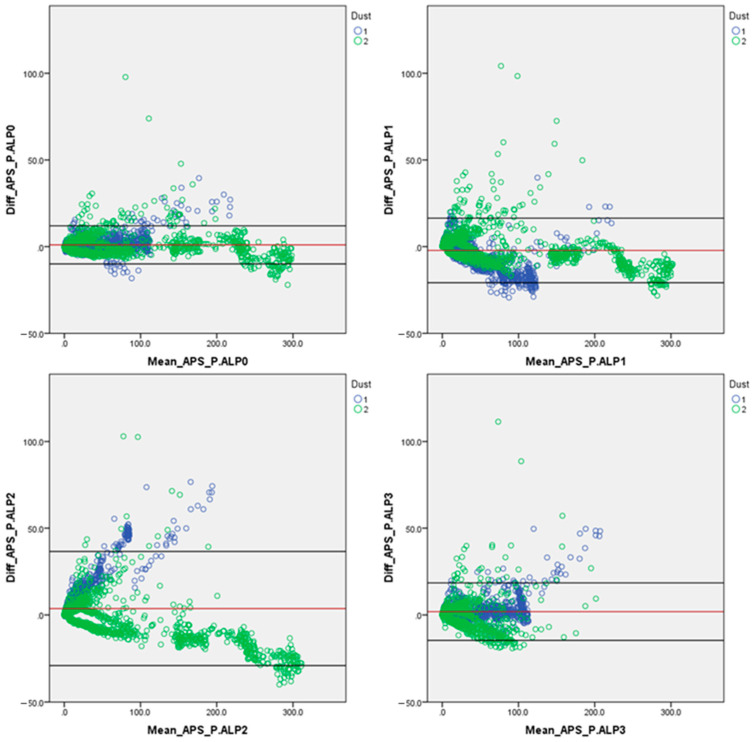
Bland–Altman plots for the four prototypes’ acquired data, corrected (TWA) by the data collected by the TEOM, plotted against the reference device (APS); both X and Y axes are expressed in [µg/m^3^]. In blue are highlighted the data referring to GrMD and in green are highlighted the data referring to GoMD. The solid red line highlights the mean error between the compared instruments, and the two solid black lines indicate the upper and the lower 95% confidence intervals.

**Figure 3:**
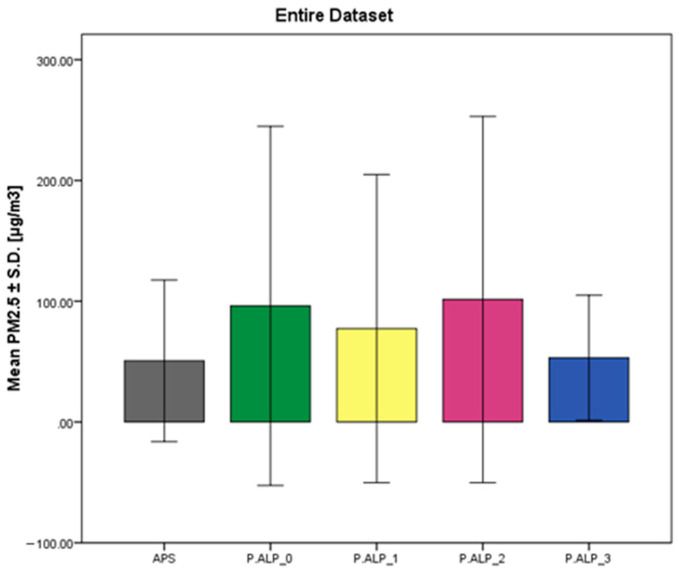
Average values reported in [µg/m^3^] ± S.D. of the reference device (APS) and the four P.ALP prototypes considering the entire set of PM2.5 concentrations.

**Figure 4:**
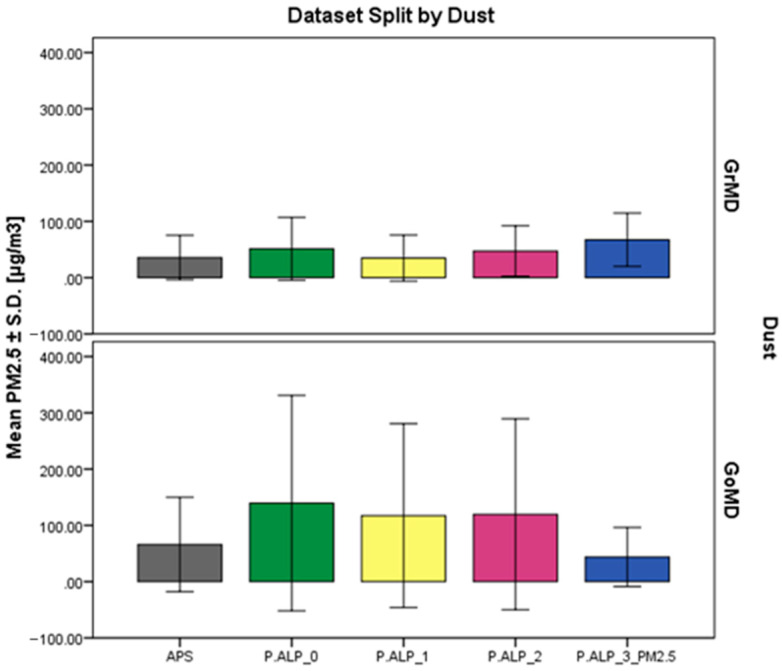
Bar chart of the mean concentration values reported in [µg/m^3^] ± S.D. of the four P.ALP prototypes and the reference instrument (APS) concerning the set of data split by the two dusts used (GrMD and GoMD).

**Figure 5:**
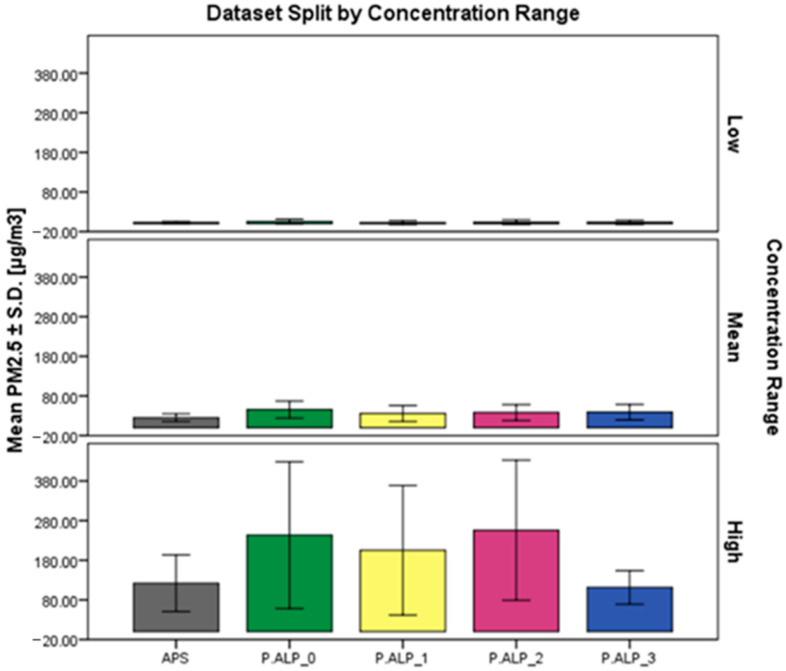
Bar chart for the mean concentration values reported in [µg/m^3^] ± S.D. of the four P.ALP prototypes and the reference instrument (APS) concerning the set of data split by the three different concentration ranges evaluated: (i) low (<9.39 µg/m^3^); (ii) mean (9.39 < x < 45.97 µg/m^3^); (iii) high (>45.97 µg/m^3^).

As expected, correcting the P.ALPs data through the equal approach adopted for the APS data (introducing correction factors obtained by TWA TEOM’s data), the performances of the prototypes remarkably improved because, following this approach, the two techniques compared were, on average, made to align with the same value (TWA TEOM’s value). Table 8 reports the average error of the P.ALPs that, in this case, are very close to zero, which means that the two instruments compared are, on average, close to perfect. After the performance evaluation using non-corrected P.ALPs’ data, this analysis was conducted to highlight that even our prototype can have similar outcomes as a laboratory instrument (APS—which costs more than one order of magnitude more than the P.ALP) if a post-correction factor is applied (Figure 2).

### 4.7. Strengths and Limitations of the Study

The reliability of the prototypes represented the main limitation of this study since the P.ALPs’ improvement is still ongoing. In fact, this affected the very first sessions of testing because, in a few cases, we could not collect usable data. Another important limitation of this study was the impossibility to stress the sensors with very-high relative humidity exposures. This was due to technical issues (i.e., moisture inside the pipes that transports the air into the radioactive charger neutralizer of the dust chamber) that occurred during the testing sessions, especially at relative humidities higher than 60%. Moreover, the fact that some missing data sessions occurred during the very first days of the experiments did not allow us to compare two sets of data acquired under exactly the same exposure conditions, but with different dusts. Furthermore, it should be made clear that the design of the conducted test sessions involved testing the sensors over a wide range of dust concentrations, generating dust peaks and subsequently cleaning the chamber environment. Therefore, the SD values of PM concentrations—which might appear unusually high—are indicative of a particularly variable trend in concentrations, generated specifically for the study. Lastly, since the data regarding RH and T were collected during each testing session and evidenced compatible circumstances with the nominal operating interval of the prototype’s PM sensor (Plantower PMS5003; usage suggested T interval: −10~+60 °C; usage suggested RH interval: 0~99%), they were not evaluated in this manuscript. Despite this, it is widely recognized in the scientific literature [28,34,35,36,37] that, concerning light-scattering PM sensors, both T and RH are interfering factors. Even though it is foreseen that these interferences could arise at higher RH and T values than the ones achieved in this project, their influence on the P.ALP’s performance will be further investigated in future studies. Future studies will also inquire into the potential sources of error, especially regarding the P.ALP’s data overestimations at high PM_2.5_ concentrations (>45.97 µg/m^3^). Dusts used in this study cannot be considered representative of airborne particles in ambient/urban air. This issue was beyond the aims of this study. Since urban dust is composed of a wide variety of different dusts generated by various sources, a dedicated study on the evaluation of the P.ALP’s performance in real working conditions has already been published and cited [25] in the present work. Hopefully, in future studies, it will also be possible to expand the experimental design to include the use of other reference instruments and dusts (and overall increase the number of test days and data available) to consolidate the analysis of the P.ALP’s performance.

Nevertheless, with the abovementioned limitations and a previous in-field assessment of the device [25], this is the very first in-lab study able to evaluate the performances of the P.ALP prototype and deeply understand the wide range of possibilities created by these technologies. The most important strength of this study is the fact that, thanks to the calm-air dust chamber used, we were able to stress the prototypes under a very wide range of operational situations, but always under controlled conditions. Despite the availability in the scientific literature of several papers focusing on the evaluation of the performances of the Plantower PMS5003 PM sensor [38,39], an assessment of the whole monitoring system, especially if it is a new prototype, must be conducted and provided because the sensor’s performance could vary depending on different factors (e.g., power supply, hardware components, pin linkage, and case). Thanks to the P.ALP project, the assessment of the device itself allowed us to provide a fully open source approach, ranging from the design to in-field and in-lab performance assessments, and a tool that can be (i) a good base for further improvements, (ii) adopted in several applications, and (iii) customized following specific needs. From these aspects, the great potential of these technologies has been highlighted and their usefulness in the air-quality monitoring field has been successfully proved.

### 4.8. Future Developments

The P.ALP monitoring system represents a promising starting point for the development of a multipollutant monitoring device. The present work is the second fundamental step of a more comprehensive assessment of the P.ALP’s performance; in fact, a previous publication [24] reported the conceptualization and the development of the P.ALP. To investigate the prototypes’ performance in non-regulated conditions in four miscellaneous microenvironments, focusing on their assessment in real working circumstances and during a vast range of PM_2.5_ concentrations, an in-field monitoring campaign has already been launched and a further manuscript on the prototype’s performances in real working conditions has already been published [25]. Overall, the results from the present study, paired with the previous in-filed study [25], provide a fully comprehensive characterization of the P.ALP’s performance in different applicability scenarios. Looking at the wide range of low-cost technologies, it will be possible to select further sensors that could be integrated into our prototype setting. Moreover, improvements in data transmission technologies (e.g., WSN—Wireless Sensor Networks and IoT—Internet of Things) will be a useful upgrade to make the P.ALP the most useful as possible in several study design strategies. In this direction, every further improvement will be a step forward in low-cost technologies’ applications in sciences assessing exposure.

## 5. Conclusions

The early outcomes suggest that the P.ALP needs to be calibrated to improve its performance, but it can follow the concentration trends with reasonable accuracy, even without any post-correction factor applications. Due to the very-low cost per unit (USD 150), if compared to the most expensive Direct Reading Instruments or even more expensive reference instruments, P.ALPs can be improved and adopted in a very wide range of applications and study designs. Some of the main benefits of the P.ALP are the reduced form factor, the low power consumption, and cost, as well as the high spatiotemporal resolution of the data acquired. The novelty of the P.ALP project is mostly found in the ‘open source’ philosophy adopted, since all the know-how concerning the hardware, software, and construction of the prototype is published in a step-by-step user manual [24], which allows anyone interested to build, improve, and customize their own monitoring device. Further tests and analyses have already been conducted [25] to elucidate the P.ALP’s performance, even in different real exposure scenarios and make it as customizable as possible (e.g., introducing new sensors for different pollutants), depending on the users’ needs.

## Figures and Tables

**Table 1:** Summary of the data-collecting sessions plan. Date: testing day in the year 2022; Testing Day: r of the day of the experiment in the dust chamber lab; Test n.: number of conducted specific tests; Planned RH: indication of RH% level, which is expected when conducting the test. Planned Test: brief description of the test to be conducted. The terms used to indicate concentrations (‘very low’, ‘low’, ‘mid-range’, ‘high’, and ‘very high’) are qualitative terms that generically indicate the expected conditions in the booth. With this experimental design, it is not possible to quantitatively define a priori precisely the level of concentration within the cabin.

Testing Day (Date)	Dust	Test n.	Planned RH	Planned Test
1 (23 September 2022)	1	1	20%	Sensitivity test
		2	25%	Low concentrations
2 (26 September 2022)	1	3	50%	Low concentrations
		4	75%	Low concentrations
3 (30 September 2022)	1	5	50%	Steady-state increasing concentrations
4 (4 October 2022)	1	6	50%	Very-high concentrations
		7	50%	Very-low concentrations
		8	Random	Random spikes
5 (5 October 2022)	1	9	Random	Random spikes
6 (11 October 2022)	2	10	25%	Sensitivity test
		11	25%	Low concentrations
7 (13 October 2022)	2	12	50%	Low concentrations
		13	75%	Low concentrations
8 (14 October 2022)	2	14	50%	Low steady-state concentrations
		15	50%	Mid-range steady-state concentrations
		16	50%	High steady-state concentrations
9 (17 October 2022)	2	17	50%	Very-low concentrations
		18	50%	Mid-range concentrations
10 (18 October 2022)	2	19	Random	Random spikes
		20	Random	Random spikes

**Table 2:** Overview of the data collection sessions. Testing Day: day (incremental number) of experiment in the dust-chamber lab; Test: number of conducted specific tests; Duration: minutes of testing; PM_2.5_ Conc.: value of PM_2.5_ concentration inside the dust chamber acquired by the device assumed as the reference (APS), reported in µg/m^3^; RH: relative humidity inside the dust chamber during the specific test; T: average temperature inside the chamber during the specific test.

Testing Day	Dust	Test	Duration [min]	PM_2.5_ Conc. [µg/m^3^]			RH [%]		T [°C]
				Min.	Mean	Max.	Min.	Max.	Mean
1	1	1	105	0.04	1.48	8.94	24.7	27.8	29.2
		2	167	0.12	2.80	9.36	22.5	45.7	30.0
2	1	3	213	0.06	3.07	9.24	29.1	57.4	30.9
		4	177	0.18	12.73	42.67	22.5	47.5	30
3	1	5	341	9.39	29.11	231.20	28.2	57.4	31.3
4	1	6	78	58.41	102.19	212.93	42.6	47	27.7
		7	130	46.13	62.72	95.63	43.8	45.3	30.1
		8	129	58.38	93.15	113.83	34.5	45.7	30.6
5	1	9	129	46.21	82.12	109.78	40.1	46	29.9
6	2	10	142	0.19	0.27	0.43	28.1	39.5	26.8
		11	179	0.12	3.89	9.35	25.1	58	29.8
7	2	12	216	0.10	9.50	45.73	25.1	57.4	30.4
		13	179	9.54	25.33	45.97	25.4	57.6	31
8	2	14	101	22.78	29.89	34.58	41.8	42.9	28.7
		15	111	9.42	23.82	43.73	26.0	43.1	28.9
		16	89	9.47	22.40	45.84	36.6	44.2	31.6
9	2	17	98	9.83	85.75	208.62	24.9	45.7	29.9
		18	146	48.41	131.50	179.36	32.6	57.9	29.2
10	2	19	137	174.54	240.07	297.34	41.1	41.7	31.8
		20	122	46.08	144.85	298.64	35.2	40.7	30

**Table 3:** PM_2.5_ concentrations, expressed in [µg/m^3^], acquired with different monitoring devices. Valid N: number of datapoints used for statistical analysis; <LOD: number of datapoints lower than the LOD of the considered instrument; Min.: minimum collected value; Mean: mean value of the data acquired by the devices; Median: median value of the device; Max.: maximum collected value; S.D.: standard deviation.

Device	Valid N	<LOD	PM_2.5_ Conc. [µg/m^3^]				
			Min.	Mean	Median	Max.	S.D.
APS	2802	0	0.04	50.69	25.12	297.34	66.88
P.ALP_0	2989	365	0.00	96.18	42.40	705.50	148.68
P.ALP_1	2942	771	0.00	77.35	30.65	588.40	127.56
P.ALP_2	2031	379	0.00	101.44	38.50	618.30	151.65
P.ALP_3	2027	376	0.00	53.16	40.00	352.20	51.78

**Table 4:** Regression coefficients between the prototypes. R: Pearson correlation parameter; R^2^: determination parameter; Q: intercept value; m: slope value; SE: standard error value; C: comparable devices (following Watson et al.’s (1998) criteria [27]; highlighted in green, if positive); MP: mutually predictable devices (following Watson et al.’s (1998) criteria [27]; highlighted in green, if positive).

Devices Compared	Regression Model					Watson et al.’s Criteria [27]	
	R	R^2^	Q	m	SE	C	MP
P.ALP_0 vs. P.ALP_1	0.998	0.995	−3.685	0.85	0.195	Yes	No
P.ALP_0 vs. P.ALP_2	0.998	0.995	−6.014	0.883	0.288	Yes	No
P.ALP_0 vs. P.ALP_3	0.992	0.983	−1.874	0.829	0.217	Yes	No
P.ALP_1 vs. P.ALP_2	0.999	0.998	−2.265	1.037	0.182	Yes	No
P.ALP_1 vs. P.ALP_3	0.992	0.984	−0.198	1.03	0.21	Yes	Yes
P.ALP_2 vs. P.ALP_3	0.989	0.979	0.954	1.008	0.243	Yes	No

**Table 5:** Regression coefficients between the four prototypes and the APS. R: Pearson correlation parameter; R^2^: determination parameter; Q: intercept value; m: slope value; SE: standard error value; C: comparable devices (following Watson et al.’s (1998) criteria [27]; highlighted in green, if positive); MP: mutually predictable devices (following Watson et al.’s (1998) criteria [27]; highlighted in green, if positive).

Devices Compared	Regression Model					Watson et al. Criteria [27]	
	R	R^2^	Q	m	SE	C	MP
P.ALP_0 vs. APS	0.979	0.959	−12.061	2.227	0.73	Yes	No
P.ALP_1 vs. APS	0.972	0.945	−13.03	1.892	0.728	Yes	No
P.ALP_2 vs. APS	0.973	0.946	−13.78	1.989	1.065	Yes	No
P.ALP_3 vs. APS	0.928	0.861	4.807	1.28	0.652	Yes	No

**Table 6:** Application of the US EPA’s Air-Sensor Guidebook criteria to position the P.ALPs in their usage domains. N: number of collected data; Mean: mean of the whole dataset considered in this assessment; SD: standard deviation value; CV: coefficient of variation value; CVdiff.: differential CV between one of the reference instruments (APS) and the four different P.ALPs; MNB: mean normalized bias; Application Tier: outcome of the EPA’s criteria adoption (highlighted in green), in the case of not being able to place the prototypes in the less limited tier (Tier I), a “Failed” note was reported.

Devices	PM_2.5_ [µg/m^3^]			EPA Criteria			
	N	Mean	SD	Cv	CVdiff.	MNB	Application Tier
P.ALP_0	2989	96.18	148.68	1.5	0.23	0.90	Failed
P.ALP_1	2989	77.35	127.56	1.6	0.33	0.53	Failed
P.ALP_2	2989	101.44	151.65	1.5	0.18	1.00	Failed
P.ALP_3	2989	53.16	51.78	1.0	−0.35	0.05	Tier I
APS	2989	50.69	66.88	1.3	-	-	-

**Table 7:** Statistics of the average error trends adopted for building the Bland–Altman plots, reported in Figure 1. Mean: mean of the whole dataset analyzed; SD: standard deviation value.

Devices Compared	PM_2.5_ Average Error [µg/m^3^]		PM_2.5_ Confidence Interval [µg/m^3^]	
	Mean	SD	Upper 95%	Lower 95%
APS vs. P.ALP_0	−50.11	87.61	121.61	−221.84
APS vs. P.ALP_1	−31.40	67.25	100.41	−163.21
APS vs. P.ALP_2	−46.55	83.27	116.65	−209.76
APS vs. P.ALP_3	−15.95	22.08	27.33	−59.23

## Data Availability

Data are contained within the article and Supplementary Materials.

## Supplementary Materials

The following are available online at https://www.mdpi.com/article/10.3390/s24185915/s1, Table S1: PM_2.5_ concentrations acquired with different monitoring devices split by concentration range; Table S2: PM_2.5_ concentrations acquired with different monitoring devices split by dust; Table S3: Regression parameters between P.ALPs splitting the dataset by concentration range; Table S4: Regression parameters between P.ALPs splitting the dataset by dust; Table S5: Regression parameters between the four P.ALPs and the APS splitting the dataset by concentration range; Table S6: Regression parameters between the four P.ALPs and the APS splitting the dataset by dust; Table S7: Application of the EPA Air Sensor Guidebook guidelines to place the P.ALPs prototype in their application field splitting the dataset by concentration range; Table S8: Application of the EPA Air Sensor Guidebook guidelines to place the P.ALPs prototype in their application field splitting the dataset by dust; Table S9: Application of the EPA Air Sensor Guidebook guidelines to place the P.ALPs prototype in their application field splitting the dataset by dust and by concentration range; Figure S1: Bland-Altman plots of the four P.ALPs acquired data expressed in [µg/m^3^], focused on PM_2.5_ low concentrations (Concentration Range: 1), plotted against the reference instrument (APS); Figure S2: Bland-Altman plots of the four P.ALPs acquired data expressed in [µg/m^3^], focused on PM_2.5_ mean concentrations (Concentration Range: 2), plotted against the reference instrument (APS); Figure S3: Bland-Altman plots of the four P.ALPs acquired data expressed in [µg/m^3^], focused on PM_2.5_ high concentrations (Concentration Range: 3), plotted against the reference instrument (APS).
