# Supplementary material for: First in-Lab Testing of a Cost-Effective Prototype for PM2.5 Monitoring: The P.ALP Assessment
Source: Sensors (Basel). 2024 Sep 12;24(18):5915. doi: 10.3390/s24185915 (PMC11436052; doi:10.3390/s24185915)
Supplement: Supplementary file 1 [file sensors-24-05915-s001.zip › sensors-3069663-supplementary.pdf]

Supplementary Materials: First in-Lab Testing of a Cost-Effective Prototype for $PM_{2.5}$ Monitoring: The P.ALP Assessment

Giacomo Fanti [1,*], Francesca Borghi [3], Cody Wolfe [2], Davide Campagnolo [1], Justin Patts [2], Andrea Cattaneo [1], Andrea Spinazzè [1], Emanuele Cauda [2] and Domenico Maria Cavallo [1]

Table 1. $PM_{2.5}$ concentrations acquired with different monitoring devices split by concentration range.

$PM_{2.5}$ –Dataset split by Concentration Range (μg/m^3)										
**Device**	**CR**	**N**	**Valid N**	**Missing**	**<LOD**	**Min.**	**Mean**	**Median**	**Max.**	**S.D.**
APS	1	1041	924	117	0	0.04	2.93	1.98	9.38	2.83
	2	974	926	48	0	9.39	25.08	24.76	45.98	9.89
	3	974	952	22	0	46.08	121.95	95.74	297.34	71.64
P.ALP_0	1	1041	1041	0	365	0	5.60	3.50	29.50	5.83
	2	974	974	0	0	5.10	45.58	44.00	116.80	21.34
	3	974	974	0	0	42.60	243.58	155.80	705.50	185.21
P.ALP_1	1	1041	1041	0	765	0	2.48	0	26.00	5.06
	2	974	974	0	6	0.20	35.66	33.90	104.40	20.22
	3	974	927	47	0	12.60	205.25	121.50	588.40	163.45
P.ALP_2	1	1041	586	455	375	0	3.66	0.50	23.70	5.98
	2	974	761	213	4	0.20	37.98	36.00	103.70	20.09
	3	974	684	290	0	13.80	255.81	169.85	618.30	176.97
P.ALP_3	1	1041	581	460	375	0	3.43	0.10	23.00	5.59
	2	974	765	209	1	1.60	38.97	37.80	105.90	19.45
	3	974	681	293	0	17.40	111.52	107.50	352.20	42.28

CR: concentration range investigated where (i) "1" represents low concentrations, (ii) "2" represents mean concentrations and (iii) "3" represents high concentrations; N: number of datapoint monitored; Valid N: number of datapoint used for statistical analysis; Missing: number of missing values; <LOD: number of datapoints lower than the LOD of the instrument; Min.: minimum; Mean: mean value of the data collected by the instrument; Median: median value of the instrument; Max.: maximum; S.D.: standard deviation.

Table 2. $PM_{2.5}$ concentrations acquired with different monitoring devices split by dust.

$PM_{2.5}$ –Dataset split by Dust (μg/m³)										
**Device**	**Dust**	**N**	**Valid N**	**Missing**	**<LOD**	**Min.**	**Mean**	**Median**	**Max.**	**S.D.**
APS	1	1469	1423	46	0	0.04	35.76	19.84	231.20	39.46
	2	1520	1379	141	0	0.10	66.09	27.21	297.34	83.76
P.ALP_0	1	1469	1469	0	237	0	51.34	27.90	311.30	55.83
	2	1520	1520	0	128	0	139.51	50.70	705.50	191.44
P.ALP_1	1	1469	1422	47	455	0	34.79	18.20	202.40	40.84
	2	1520	1520	0	316	0	117.17	40.95	588.40	163.28
P.ALP_2	1	1469	512	957	66	0	47.35	37.35	193.00	44.94
	2	1520	1519	1	313	0	119.67	38.60	618.30	169.57
P.ALP_3	1	1469	807	662	70	0	67.32	66.30	231.10	47.34
	2	1520	1220	300	306	0	43.79	30.20	352.20	52.48

Dust: dust investigated where (i) "1" represents grant mine dust and (ii) "2" represents gold mine dust; N: number of datapoint monitored; Valid N: number of datapoint used for statistical analysis; Missing: number of missing values; <LOD: number of datapoints lower than the LOD of the instrument; Min.: minimum; Mean: mean value of the data collected by the instrument; Median: median value of the instrument; Max.: maximum; S.D.: standard deviation.

Table 3. Regression parameters between P.ALPs splitting the dataset by concentration range.

**Devices Compared**	**CR**	**Regression Model**					**Watson et al. Criteria [27]**	
		**R**	**R^2**	**Q**	**m**	**SE**	**C**	**MP**
**P.ALP_0 vs. P.ALP_1**	1	0.924	0.854	-2.008	0.801	0.083	Yes	No
	2	0.962	0.925	-5.894	0.912	0.419	Yes	No
	3	0.996	0.992	-9.255	0.864	0.77	Yes	No
P.ALP_0 vs. P.ALP_2	1	0.949	0.900	-2.491	0.854	0.115	Yes	No
	2	0.965	0.930	-6.7	0.906	0.484	Yes	No
	3	0.996	0.992	-16.949	0.908	1.125	Yes	No
P.ALP_0 vs. P.ALP_3	1	0.942	0.888	-2.288	0.719	0.115	Yes	No
	2	0.976	0.953	-5.107	0.89	0.386	Yes	No
	3	0.971	0.944	4.954	0.784	1.071	Yes	No
P.ALP_1 vs. P.ALP_2	1	0.989	0.979	0.029	0.943	0.042	Yes	Yes
	2	0.984	0.967	-0.624	0.972	0.289	Yes	Yes
	3	0.998	0.996	-5.164	1.045	0.777	Yes	No
P.ALP_1 vs. P.ALP_3	1	0.976	0.953	0.062	0.867	0.059	Yes	Yes
	2	0.980	0.960	1.468	0.941	0.309	Yes	No
	3	0.976	0.953	9.666	0.916	0.976	Yes	No
P.ALP_2 vs. P.ALP_3	1	0.976	0.953	0.08	0.909	0.059	Yes	Yes
	2	0.980	0.961	2.69	0.952	0.3	Yes	No
	3	0.956	0.914	15.019	0.913	1.853	Yes	No

CR: concentration range investigated where (i) "1" represents low concentrations, (ii) "2" represents mean concentrations and (iii) "3" represents high concentrations; R: Pearson correlation coefficient; R^2: Determination coefficient; Q: Intercept; m: Slope; SE: Standard Error; C: Comparable (following the Watson et al., 1198 criteria [27]); MP: Mutually Predictable (following the Watson et al., 1198 criteria [27]). In green are highlighted the comparisons that satisfy the Watson et al. Criteria [27] of comparability and/or mutual predictivity.

Table 4. Regression parameters between P.ALPs splitting the dataset by dust.

Devices Compared	Dust	Regression Model					Watson et al. Criteria [27]	
		R	R^2	Q	m	SE	C	MP
**P.ALP_0 vs. P.ALP_1**	1	0.994	0.998	-1.576	0.756	0.16	Yes	No
	2	0.998	0.996	-1.6	0.851	0.319	Yes	No
P.ALP_0 vs. P.ALP_2	1	0.994	0.989	-0.957	0.702	0.308	Yes	No
	2	0.999	0.997	-3.81	0.885	0.273	Yes	No
P.ALP_0 vs. P.ALP_3	1	0.991	0.982	-1.829	0.829	0.394	Yes	No
	2	0.991	0.983	-1.895	0.828	0.263	Yes	No
P.ALP_1 vs. P.ALP_2	1	0.998	0.995	-0.28	0.963	0.186	Yes	Yes
	2	0.999	0.998	-1.963	1.037	0.299	Yes	No
P.ALP_1 vs. P.ALP_3	1	0.997	0.995	-1.916	1.125	0.212	Yes	No
	2	0.994	0.989	-0.436	0.981	0.207	Yes	Yes
P.ALP_2 vs. P.ALP_3	1	0.995	0.990	-0.739	1.137	0.335	Yes	Yes
	2	0.993	0.986	0.854	0.968	0.234	Yes	No

Dust: dust investigated where (i) “1” represents grant mine dust and (ii) “2” represents gold mine dust; R: Pearson correlation coefficient; R^2: Determination coefficient; Q: Intercept; m: Slope; SE: Standard Error; C: Comparable (following the Watson et al., 1198 criteria [27]); MP: Mutually Predictable (following the Watson et al., 1198 criteria [27]). In green are highlighted the comparisons that satisfy the Watson et al. Criteria [27] of comparability and/or mutual predictivity.

Table 5. Regression parameters between the four P.ALPs and the APS splitting the dataset by concentration range.

Devices Compared	CR	Regression Model					Watson et al. Criteria [27]	
		R	R^2	Q	m	SE	C	MP
**P.ALP_0 vs. APS**	1	0.858	0.735	0.451	1.852	0.149	No	No
	2	0.825	0.68	1.302	1.777	1.081	No	No
	3	0.973	0.947	-62.305	2.531	2.746	Yes	No
P.ALP_1 vs. APS	1	0.69	0.476	-1.051	1.295	0.182	No	No
	2	0.714	0.51	-0.802	1.461	1.27	No	No
	3	0.963	0.927	-58.039	2.161	2.898	Yes	No
P.ALP_2 vs. APS	1	0.749	0.561	-0.849	1.578	0.281	No	No
	2	0.694	0.482	1.381	1.421	1.501	No	No
	3	0.953	0.907	-53.838	2.20	4.379	Yes	No
P.ALP_3 vs. APS	1	0.759	0.577	-0.757	1.483	0.259	No	No
	2	0.762	0.581	0.298	1.503	1.304	No	No
	3	0.748	0.56	26.946	1.037	3.139	No	No

CR: concentration range investigated where (i) “1” represents low concentrations, (ii) “2” represents mean concentrations and (iii) “3” represents high concentrations; R: Pearson correlation coefficient; R^2: Determination coefficient; Q: Intercept; m: Slope; SE: Standard Error; C: Comparable (following the Watson et al., 1198 criteria [27]); MP: Mutually Predictable (following the Watson et al., 1198 criteria [27]). In green are highlighted the comparisons that satisfy the Watson et al. Criteria [27] of comparability and/or mutual predictivity.

Table 6. Regression parameters between the four P.ALPs and the APS splitting the dataset by dust.

Devices Compared	Dust	Regression Model					Watson et al. Criteria [27]	
		R	R^2	Q	m	SE	C	MP
**P.ALP_0 vs. APS**	1	0.995	0.99	1.223	1.419	0.197	Yes	No
	2	0.997	0.994	-3.586	2.342	0.532	Yes	No
P.ALP_1 vs. APS	1	0.998	0.976	-0.548	1.067	0.227	Yes	Yes
	2	0.994	0.988	-4.517	1.992	0.62	Yes	No
P.ALP_2 vs. APS	1	0.982	0.965	1.972	0.965	0.535	Yes	Yes
	2	0.994	0.988	-7.047	2.07	0.655	Yes	Yes
P.ALP_3 vs. APS	1	0.986	0.972	1.254	1.154	0.491	Yes	Yes
	2	0.954	0.91	0.346	1.741	0.664	Yes	No

Dust: dust investigated where (i) "1" represents grant mine dust and (ii) "2" represents gold mine dust; R: Pearson correlation coefficient; R^2: Determination coefficient; Q: Intercept; m: Slope; SE: Standard Error; C: Comparable (following the Watson et al., 1198 criteria [27]); MP: Mutually Predictable (following the Watson et al., 1198 criteria [27]). In green are highlighted the comparisons that satisfy the Watson et al. Criteria [27] of comparability and/or mutual predictivity.

Table 7. Application of the EPA Air Sensor Guidebook guidelines to place the P.ALPs prototype in their application field splitting the dataset by concentration range.

Devices	CR	$PM_{2.5}$ [μg/m³]			EPA criteria			
		N	Mean	SD	CV	CVdiff.	MNB	Application Tier
**P.ALP_0**	1	1041	5.60	5.83	1.0	0.08	0.91	Failed
	2	974	45.58	21.34	0.5	0.07	0.82	Tier I
	3	974	243.58	185.21	0.8	0.17	1.00	Failed
P.ALP_1	1	1041	2.48	5.06	2	1.08	-0.15	Failed
	2	974	35.66	20.22	0.6	0.17	0.42	Failed
	3	974	205.25	163.45	0.8	0.21	0.68	Failed
P.ALP_2	1	1041	3.66	5.98	1.6	0.67	0.25	Failed
	2	974	37.98	20.09	0.5	0.13	0.51	Failed
	3	974	255.81	176.97	0.7	0.10	1.10	Failed
P.ALP_3	1	1041	3.43	5.59	1.6	0.66	0.17	Failed
	2	974	38.97	19.45	0.5	0.10	0.55	Failed
	3	974	111.52	42.28	0.4	-0.21	-0.09	Tier II & IV
APS	1	1041	2.93	2.83	1	-	-	-
	2	974	25.08	9.89	0.4	-	-	-
	3	974	121.95	71.64	0.6	-	-	-

CR: concentration range investigated where (i) "1" represents low concentrations, (ii) "2" represents mean concentrations and (iii) "3" represents high concentrations; N: number of data; Mean: mean of the entire dataset utilized in this evaluation; SD: standard deviation; CV: coefficient of variation; CVdiff.: differential coefficient of variation between the CV of the reference grade instrument APS and the four different prototypes. MNB: mean normalized bias; Application Tier: the result of the application of the EPA criteria (highlighted in green), in case of impossibility to categorize the prototypes even in the less stringent tier (tier I) the "Failed" note was adopted.

Table 8. Application of the EPA Air Sensor Guidebook guidelines to place the P.ALPs prototype in their application field splitting the dataset by dust.

Devices	Dust	$PM_{2.5}$ [μg/m³]			EPA criteria			
		N	Mean	SD	CV	CVdiff.	MNB	Application Tier
**P.ALP_0**	1	1469	51.34	55.83	1.1	-0.02	0.44	Tier I
	2	1520	139.51	191.44	1.4	0.10	1.11	Failed
P.ALP_1	1	1469	34.79	40.84	1.2	0.07	-0.03	Tier V
	2	1520	117.17	163.28	1.4	0.13	0.77	Failed
P.ALP_2	1	1469	47.35	44.94	0.9	-0.15	0.32	Tier I
	2	1520	119.67	169.57	1.4	0.15	0.81	Failed
P.ALP_3	1	1469	67.32	47.34	0.7	-0.40	0.88	Failed
	2	1520	43.79	52.48	1.2	-0.07	-0.34	Tier I
APS	1	1469	35.76	39.46	1.1	-	-	-
	2	1520	66.09	83.76	1.3	-	-	-

Dust: dust investigated where (i) "1" represents grant mine dust and (ii) "2" represents gold mine dust; N: number of data; Mean: mean of the entire dataset utilized in this evaluation; SD: standard deviation; CV: coefficient of variation; CVdiff.: differential coefficient of variation between the CV of the reference grade instrument APS and the four different prototypes. MNB: mean normalized bias; Application Tier: the result of the application of the EPA criteria (highlighted in green), in case of impossibility to categorize the prototypes even in the less stringent tier (tier I) the "Failed" note was adopted.

Table 9. Application of the EPA Air Sensor Guidebook guidelines to place the P.ALPs prototype in their application field splitting the dataset by dust and by concentration range.

Devices	Dust	CR	PM2.5 [μg/m³]			EPA criteria			
			N	Mean	SD	CV	CVdiff.	MNB	Application Tier
**P.ALP_0**	**1**	1	563	4.20	4.00	1.0	0.02	0.44	Tier I
		2	434	36.01	16.71	0.5	0.03	0.45	Tier I
		3	472	121.66	40.13	0.3	-0.03	0.44	Tier I
	2	1	478	7.25	7.09	1.0	-0.03	1.46	Failed
		2	540	53.27	21.56	0.4	0.04	1.10	Failed
		3	502	358.22	194.76	0.5	0.03	1.28	Failed
P.ALP_1	1	1	563	1.01	1.82	1.8	0.87	-0.65	Failed
		2	434	25.05	13.73	0.5	0.12	0.01	Tier III & IV
		3	472	89.49	27.97	0.3	-0.05	0.06	Tier V
	2	1	478	4.21	6.80	1.6	0.61	0.43	Failed
		2	540	44.18	20.58	0.5	0.10	0.75	Failed
		3	502	303.25	166.50	0.5	0.04	0.93	Failed
P.ALP_2	1	1	563	1.95	2.11	1.1	0.14	-0.33	Tier I
		2	434	27.07	14.11	0.5	0.09	0.09	Tier V
		3	472	99.18	31.84	0.3	-0.04	0.18	Tier III & IV
	2	1	478	4.05	6.49	1.6	0.60	0.37	Failed
		2	540	42.44	20.46	0.5	0.12	0.68	Failed
		3	502	312.60	173.74	0.6	0.04	0.99	Failed
P.ALP_3	1	1	563	2.07	2.45	1.2	0.24	-0.29	Tier II & IV
		2	434	30.76	16.24	0.5	0.10	0.24	Tier II & IV
		3	472	99.89	31.91	0.3	-0.04	0.18	Tier III & IV
	2	1	478	3.74	6.05	1.6	0.61	0.27	Failed
		2	540	42.42	19.67	0.5	0.10	0.68	Failed
		3	502	137.79	50.37	0.4	-0.15	-0.12	Tier III & IV
APS	1	1	563	2.91	2.73	0.9	-	-	-
		2	434	24.78	10.67	0.4	-	-	-
		3	472	84.35	30.72	0.4	-	-	-
	2	1	478	2.95	2.96	1.0	-	-	-
		2	540	25.32	9.20	0.4	-	-	-
		3	502	157.40	80.59	0.5	-	-	-

Dust: dust investigated where (i) "1" represents grant mine dust and (ii) "2" represents gold mine dust; CR: concentration range investigated where (i) "1" represents low concentrations, (ii) "2" represents mean concentrations and (iii) "3" represents high concentrations; N: number of data; Mean: mean of the entire dataset utilized in this evaluation; SD: standard deviation; CV: coefficient of variation; CVdiff.: differential coefficient of variation between the CV of the reference grade instrument APS and the four different prototypes. MNB: mean normalized bias; Application Tier: the result of the application of the EPA criteria (highlighted in green), in case of impossibility to categorize the prototypes even in the less stringent tier (tier I) the "Failed" note was adopted.

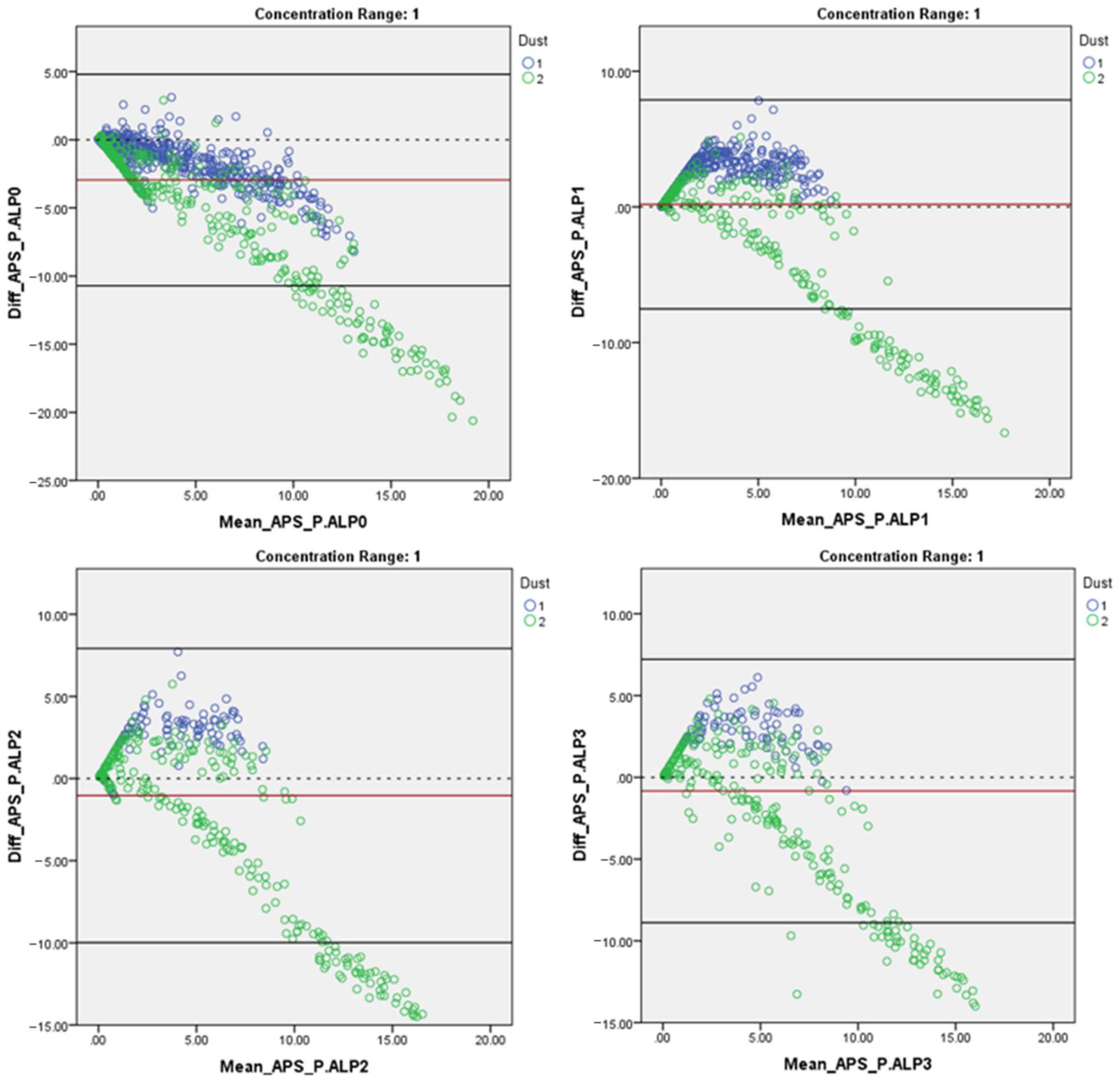

Figure 1. Bland-Altman plots of the four P.ALPs acquired data expressed in [μg/m³], focused on $PM_{2.5}$ low concentrations (Concentration Range: 1), plotted against the reference instrument (APS). In blue are highlighted the data referred to the dust 1 (GrMN) and in green are highlighted the data referred to dust 2 (GoMN). The dotted black line indicates the theoretic perfect agreement between the two compared instruments (P.ALP and APS). The solid red line represents the mean error between the compared techniques and the two solid black lines represent the upper and the lower 95% confidence intervals, respectively.

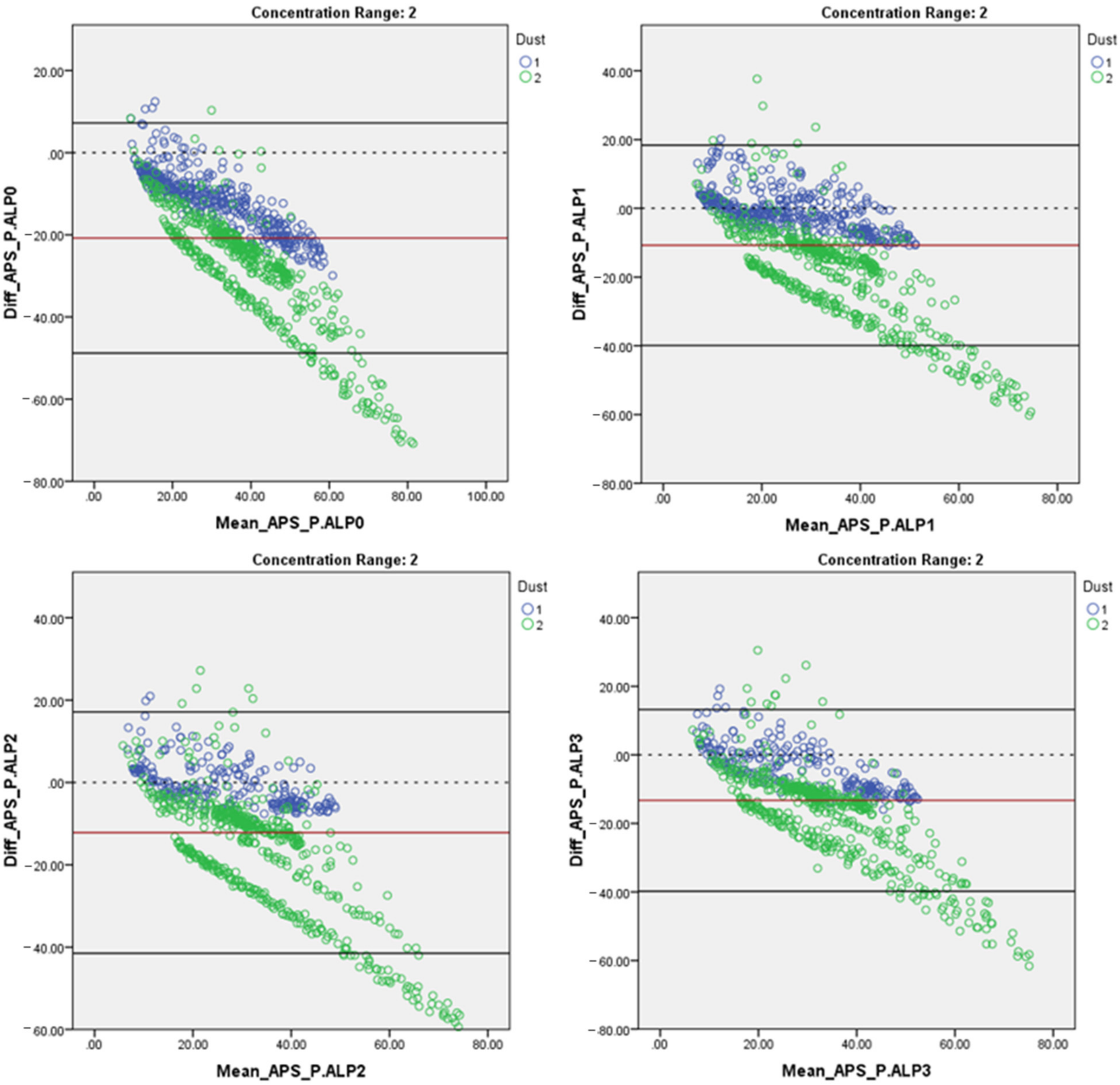

Figure 2. Bland-Altman plots of the four P.ALPs acquired data expressed in [μg/m³], focused on $PM_{2.5}$ mean concentrations (Concentration Range: 2), plotted against the reference instrument (APS). In blue are highlighted the data referred to the dust 1 (GrMN) and in green are highlighted the data referred to dust 2 (GoMN). The dotted black line indicates the theoretic perfect agreement between the two compared instruments (P.ALP and APS). The solid red line represents the mean error between the compared techniques and the two solid black lines represent the upper and the lower 95% confidence intervals, respectively.

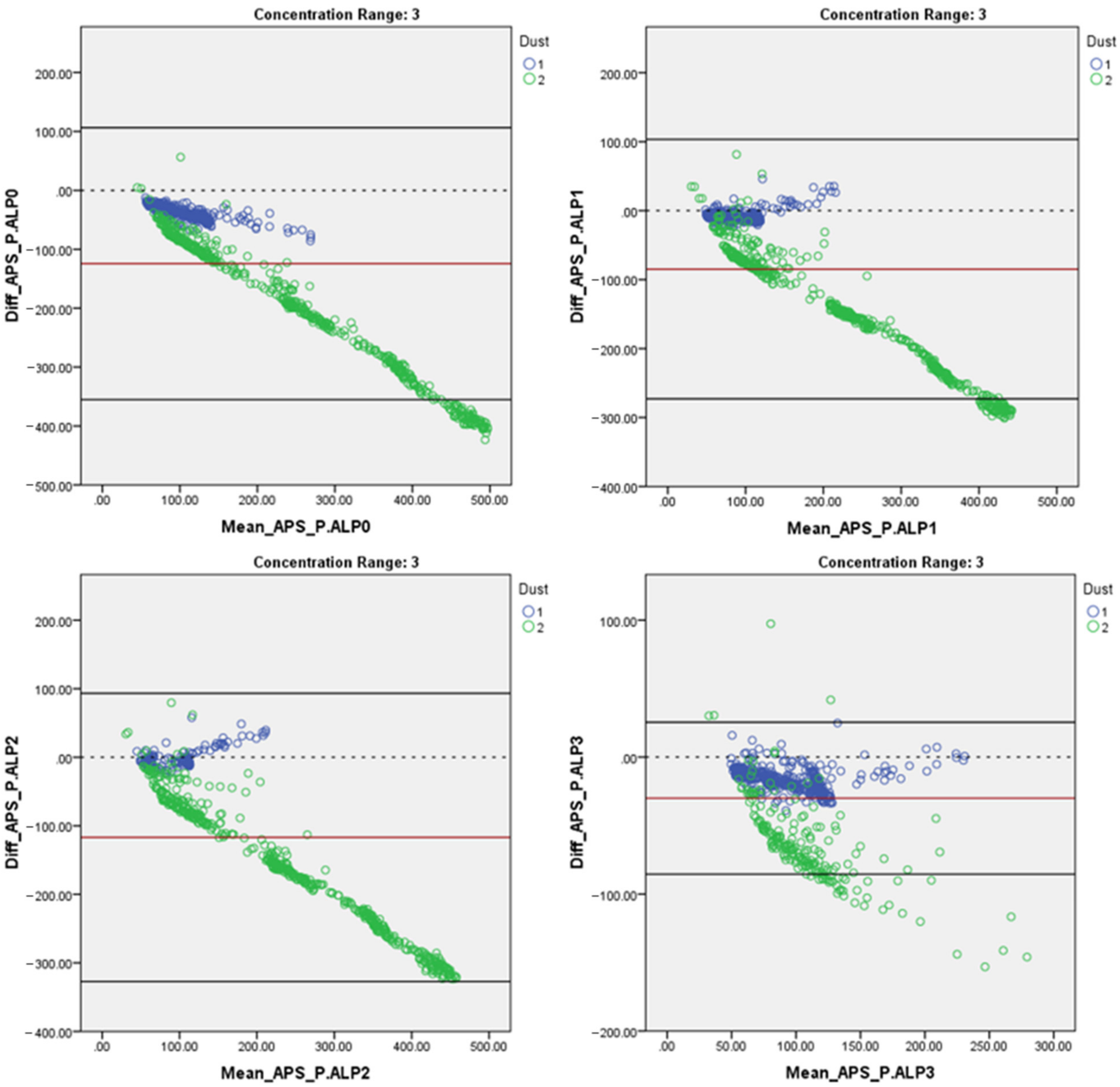

Figure 3. Bland-Altman plots of the four P.ALPs acquired data expressed in [μg/m³], focused on $PM_{2.5}$ high concentrations (Concentration Range: 3), plotted against the reference instrument (APS). In blue are highlighted the data referred to the dust 1 (GrMN) and in green are highlighted the data referred to dust 2 (GoMN). The dotted black line indicates the theoretic perfect agreement between the two compared instruments (P.ALP and APS). The solid red line represents the mean error between the compared techniques and the two solid black lines represent the upper and the lower 95% confidence intervals, respectively.
